# Construction and Validation of a New Naïve Sequestrin Library for Directed Evolution of Binders against Aggregation-Prone Peptides

**DOI:** 10.3390/ijms24010836

**Published:** 2023-01-03

**Authors:** Linnea Charlotta Hjelm, Hanna Lindberg, Stefan Ståhl, John Löfblom

**Affiliations:** Department of Protein Science, School of Engineering Sciences in Chemistry, Biotechnology and Health, KTH Royal Institute of Technology, 106 91 Stockholm, Sweden

**Keywords:** affibody, Aβ, Alzheimer’s disease, phage display, sequestrins, directed evolution

## Abstract

Affibody molecules are small affinity proteins that have excellent properties for many different applications, ranging from biotechnology to diagnostics and therapy. The relatively flat binding surface is typically resulting in high affinity and specificity when developing binding reagents for globular target proteins. For smaller unstructured peptides, the paratope of affibody molecules makes it more challenging to achieve a sufficiently large binding surface for high-affinity interactions. Here, we describe the development of a new type of protein scaffold based on a dimeric form of affibodies with a secondary structure content and mode of binding that is distinct from conventional affibody molecules. The interaction is characterized by encapsulation of the target peptide in a tunnel-like cavity upon binding. The new scaffold was used for construction of a high-complexity phage-displayed library and selections from the library against the amyloid beta peptide resulted in identification of high-affinity binders that effectively inhibited amyloid aggregation.

## 1. Introduction

Monoclonal antibody drugs have been transformative for the treatment of many different diseases during the past decades, most notably in the areas of autoimmune disorders and oncology. Although the real breakthrough in neurodegenerative disorders is still yet to be demonstrated, the recent results from the pivotal Clarity AD Phase 3 study of lecanemab is encouraging [1]. 

In parallel to the continued development and refinement of antibodies as drugs, research has also been focused on exploring non-antibody-based affinity protein as biopharmaceuticals [2,3]. Affibody molecules is a class of small (58 amino acids) affinity proteins with a three-helical structure that has been evaluated relatively extensively for in vivo diagnostics in oncology and as therapeutic drugs in both oncology and autoimmune diseases. The most advanced affibody molecule is currently tested in clinical phase III trials for treatment of different IL17A-driven autoimmune conditions and the results so far demonstrate excellent efficacy and safety [4].

In a previous effort to develop affibody molecules for amyloid beta (Aβ), involved in the pathogenesis of Alzheimer’s disease (AD), phage display technology was used for selection of binders for the peptide. Screening of hits from the selection showed that the effort was successful, and analysis of the top candidate (denoted Z_Aβ3_) revealed that the directed evolution had resulted in a new type of binder that was distinct from the original affibody protein [5]. Although the genetic sequence was similar, the three-dimensional structure and mode of binding differed quite dramatically from other reported affibody molecules. Most notably, the binder was shown to fold as a disulphide-stabilized homodimer formed by an evolved cysteine at a specific position in each subunit. Moreover, enrichment of helix-destabilizing prolines and glycines in the *N*-terminal region of the protein resulted in loss of the first helix in respective subunit [6]. Upon binding, this region co-folded with the Aβ peptide forming a four-stranded beta sheet that stabilized the interaction. In the formed complex, the aggregation-prone parts of the Aβ peptide were buried in a tunnel-like cavity, and it was later demonstrated that this resulted in inhibition of aggregation [5,6,7]. The Aβ-binder has been further engineered and a new variant with picomolar affinity (denoted Z_SYM73_) has been evaluated with encouraging results in experimental therapy studies in AD animal models [8,9,10].

Efforts have also been made to investigate whether the new type of dimeric affibody could be engineered for new specificities. Using error-prone PCR combined with phage display technology, several new binders against other aggregation-prone peptides, such as alpha synuclein, tau and islet amyloid polypeptide, where discovered, showing an almost identical structure and mode of binding as the parental amyloid-beta binder [11,12,13]. Although the affinities were modest and several binders demonstrated a relatively high degree of cross-reactivity for different peptides, the studies showed that it was possible to engineer the specificity of this new class of affinity proteins.

Encouraged by these studies, the aim here was to engineer the dimeric affibody into a scaffold protein that could be used as basis for generation of high-diversity libraries, followed by selection of new aggregation inhibitors. The scaffold was designed based on previously reported data [10,14,15], including an *N*-terminal truncation and a glycine/serine linker to form a head-to-tail dimeric fusion protein. From previously reported mutational studies and three-dimensional structures of similar dimeric affibody binders in complex with respective target peptides [10,15], eleven positions in each subunit were targeted for randomisation. Subcloning of the designed DNA library yielded a phage-displayed library with 5 × 10^9^ diversity. To assess the quality of the library, a proof-of-principle selection against Aβ was performed, yielding high-affinity binders in the low nanomolar range. The variants with highest affinity were finally analysed with respect to their effect on Aβ aggregation and the results demonstrated complete inhibition of fibril formation at a 1:1 molar ratio. Due to their capacity of sequestering aggregation-prone peptides, we propose to call this new class of binders *Sequestrins*.

## 2. Results

### 2.1. Scaffold Design and Functionality of Sequestrin Library

**Figure 1:**
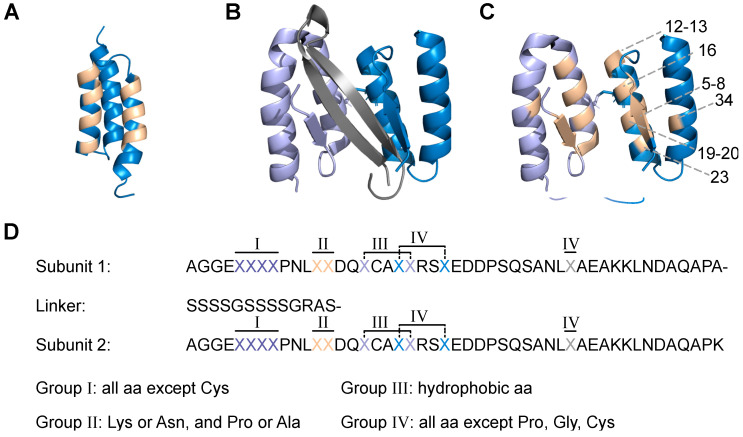
(**A**) Structure of conventional affibody molecule with randomized positions marked in beige for comparison (PDB:2B89). (**B**) Two subunits of Z_Aβ3_ [6] (light purple and blue) in complex with amyloid beta (Aβ) (grey) (PDB:2OTK). A cysteine bridge between the two subunits is shown as sticks. (**C**) The head-to-tail Sq_lib_ with the 22 randomized positions shown in beige for the two subunits. (**D**) The randomised positions in the design and with connecting linker. Each position group has a specified distribution of amino acids. The same distribution is used for both subunits.

The sequestrin (Sq) library scaffold was designed to comprise (1) a disulphide bond between the two subunits, (2) an eleven amino acid truncation of the unstructured *N*-terminus, as it has been shown to increase binding to the amyloid beta (Aβ) peptide [10,14,16], as well as (3) a flexible (S_4_G)_2_ linker between the two subunits to allow for correct folding with minimal steric hindrance (Figure 1).

The positions that were targeted for randomization constitute the binding surface of previous dimeric affibody molecules in complex with aggregation-prone peptides (Figure 1). In total, 22 positions (eleven in each subunit) were subjected to diversification. In principle, each randomized position was designed to contain the codon for the wildtype amino acid spiked with a mix of codons for a pre-determined proportion of the remaining amino acids. In 18 of the diversified positions (nine in each subunit), an average proportion of 61% of the original codon was combined with a mix of approximately 39% of mutation codons yielding on average seven mutations in the scaffold. Diversification of residues that contribute to the hydrophobic core of the complex was restricted to codons for hydrophobic amino acids in order to preserve the hydrophobic interface of the subunits. Residues that were subjected to randomization in the beta strand area were allowed to vary between all remaining amino acids except for cysteine, and thus included beta-strand stabilizing proline and glycine. The remaining diversified residues were in principle allowed to vary between any amino acid except for proline, glycine, or cysteine. In addition to these 18 randomized positions, four residues (two in each subunit) were subjected to partial randomization and allowed to vary between the original amino acid and the codon for one other amino acid to a 50:50 proportion (Figure 1D).

The two library subunits were synthesized as separate DNA oligos and amplified to contain approximately 60 overlapping bases for hybridization into one long assembled library oligo. The synthesis of the DNA was performed using a semi-conductor-based method, which provided complete freedom when selecting codons for the library and allowed randomizations with minimal biases compared to degenerate codons and error-prone PCR based approaches. The design resulted in a theoretical library size of 1.27 × 10^22^ individual candidates.

**Figure 2:**
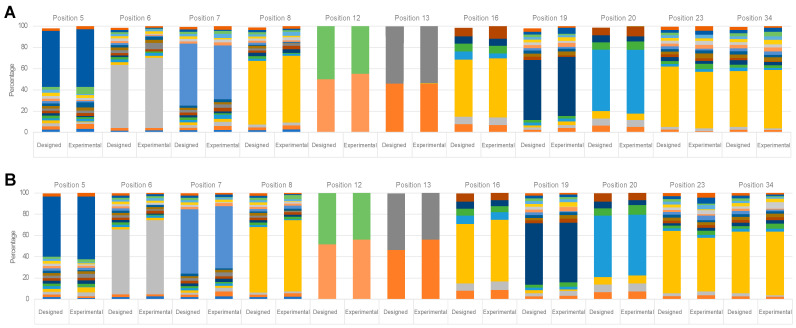
Library distribution in percentage for Sq_lib_ in the (**A**) first subunit and (**B**) second subunit at randomized positions. Generally, the design is maintained in the created library pool.

The library oligo was subcloned to a modified version of the phagemid vector pAffi1 [5] as *N*-terminal fusion to an albumin-binding domain (ABD) (Figure S1A) [17]. The resulting library (Sq_lib_) yielded 5 × 10^9^ individual clones as assessed by serial dilutions of transformed *E. coli* and had 72% sequestrins (dimers) and 24% monomers as assessed by PCR. However, a decrease in sequestrins (64%) and monomers (16%) in favour for dummy sequences (increased from 4% to 20%) was observed after phage amplification, possibly due to growth bias. To maintain a high proportion of sequestrins, the cultures were started at a higher OD_600_ (0.2 AU) than what is used in phage display selections of affibody molecules, resulting in fewer cell division cycles in the log-phase and higher proportion of phage displaying sequestrins compared to dummy phage. Moreover, likely due to the increased complexity of the sequestrin (compared to affibody molecules), a longer cultivation time was needed to reach sufficient optical density before superinfection The amino acid distribution in the library was verified by DNA sequencing (Figure 2 and Figure S2A,B). About 26% of the sequestrin sequences did not contain mutations other than from the design.

To assess the functionality of the produced phage expressing the Sq_lib_, a monoclonal sandwich ELISA was performed. Here, the *C*-terminal ABD [17] that is co-expressed with the sequestrins (Figure S1A) on the phage allowed for detection of correctly expressed construct by assessing binding of HSA in an ELISA. Correlation between a correct sequestrin sequence and an ELISA positive signal was observed.

### 2.2. Phage Selection of Sequestrins towards Amyloid Beta Peptide

After verifying the compatibility of the phage display system and sequestrins, the functionality and library design was evaluated by preforming a selection towards the Aβ_1–40_ peptide (Figure S1). The selection process was monitored by titrations of phage inputs and outputs for each cycle, showing enrichment of full sequestrin expressing clones and increase in phage titres with increased number of cycles in the selection. In the third selection cycle, an enrichment was seen in which 50% of the input phage were recovered after extensive washing. In this cycle, only full-length sequestrins were found in PCR screening of the phage eluate, indicating a successful selection.

Enrichment of target-binding clones during the selection was assessed by polyclonal phage ELISA. The phage pool showed an increased signal towards Aβ_1–40_ peptide with increasing selection rounds, correlating to phage enrichment in selection. Moreover, a shift in sequence distribution from the naïve library could be observed (Figure S2), supporting the results from titrations and the polyclonal ELISA. The clones from the output had an average of four mutations per sequestrin, compared to seven in the naïve library. Mutations were found predominantly located in the second helix in each subunit. Thirteen sequestrins among the most reoccurring clones were selected for further characterization (Table S1).

### 2.3. Expression and Cloning of Sequestrins

**Figure 3:**
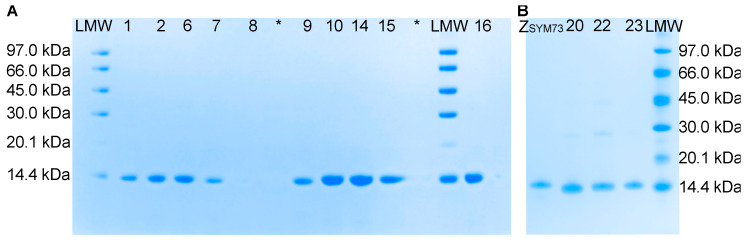
Sodium Dodecyl Sulphate–Polyacrylamide Gel (SDS-PAGE) analysis of produced sequestrins Sq_Aβ_ with 1.5 μg loaded per construct, at expected weight of 13 kDa. (**A**) Sequestrins from third and fourth cycle. LMW: low molecular weight ladder. (**B**) Sequestrins from fifth cycle. * Lanes intentionally left empty.

The thirteen clones from the selection towards the Aβ_1–40_ peptide were subcloned into a pET26b(+)-vector with a C-terminal hexa-histidine tag (further denoted as Sq_Aβ_-His_6_) for expression in *E. coli* (Table S1). The clones obtained from the fourth and fifth selection round (clones 10–23) showed somewhat improved yields during production compared to the clones from the third round. Protein purity and size was analysed by gel electrophoresis (Figure 3). A single band was observed at 14.4 kDa corresponding with the expected weight of approximately 13 kDa for all sequestrins (Table S2). The yield for Sq_Aβ8_ was very low, seen as a faint band of the expected size in the Sodium Dodecyl Sulphate–Polyacrylamide Gel (SDS-PAGE). The mass of the proteins was analysed using mass spectrometry and correlated in general well to the respective molecular weights according to sequences. However, clone Sq_Aβ8_, Sq_Aβ9_ and Sq_Aβ14_ showed a larger difference between expected and observed mass that is not explained by difference in cleavage of signal peptide during production and is likely a post translation modification (Table S2).

### 2.4. Circular Dichroism for Secondary Structure Determination of Sequestrins

Circular dichroism (CD) spectroscopy was employed to verify the secondary structure content of the new sequestrins. All the produced constructs except for Sq_Aβ8_ showed the same characteristic alpha-helical secondary structure content (Figure S3) as has previously been established for the other Aβ-binding affibody molecules such as Z_Aβ3_ [7] and Z_SYM73_ [10], indicating a similar three-dimensional structure The majority of the sequestrins show an overlap between the CD spectra obtained before and after heat-induced denaturation, indicating full refolding capability. The thermal melting point (T_m_) varied between 35 and 48 °C (Table 1), which is in the same range as previously reported Aβ-binding affibody molecules.

### 2.5. SPR-Based Biosensor Analysis of Binding to the Amyloid Beta Peptide

The affinity between the selected sequestrins and biotinylated Aβ_1–40_ was evaluated by a surface plasmon resonance (SPR)-based biosensor assay where the target antigen was immobilized on a streptavidin chip. Eleven of the sequestrins demonstrated affinities in the range of K_D_ 1–30 nM (Figure S4 and Table 1). Generally, the kinetics of the interactions were characterized by slower association compared to what is typically observed for conventional three-helical affibody molecules, likely due to structural rearrangements upon target binding as has been demonstrated previously for related dimeric peptide-binding affibody molecules [7]. Dissociation was in general very slow, approximately to 10^−5^ s^−1^ for the sequestrins with highest affinity, indicating a stable complex (Figure S4 and Table 1). Sq_Aβ8_ that was produced with low yield demonstrated a relatively weak binding. The signal from the negative control Z_Taq_ with specificity for Taq polymerase [18], was considerably lower compared to the sequestrins.

**Figure 4:**
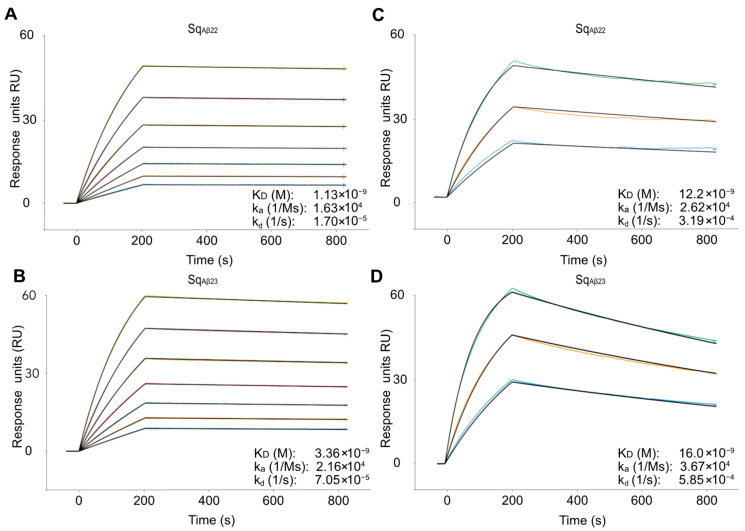
Surface plasmon resonance (SPR) sensorgram for the sequestrins Sq_Aβ22_, and Sq_Aβ23_ at (**A**,**B**) 25 °C, and (**C**,**D**) 37 °C, respectively, and their corresponding kinetic constants. The y-axis shows the relative response units (RU) and x-axis time(s). Kinetics were recorded for 900 s, with analyte injection ending at 200 s. The sequestrins were injected in duplicate in a dilution series spanning 1:1.5 steps from 342 to 30 nM for the 25 °C affinity measurement. At 37 °C the sequestrins were injected in triplicates with concentrations 300–75 nM in a 1:2 dilution series. The black lines represent the fitting of the data to calculate kinetics. The experiment was conducted with immobilized Aβ_1–40_ at a coating density of 80 RU.

The sequestrins with the highest affinity (Sq_Aβ22_ and Sq_Aβ23_ with K_D_ of 1 and 3 nM, respectively) were discovered in the output after the fifth selection cycle with more stringent washing, exemplified by clone Sq_Aβ22_ that was highly enriched in the fifth selection cycle (Figure 4) and displaying the slowest dissociation among the new binders. As expected, the off rates increased when analysed at 37 °C. Still, the affinities for Sq_Aβ22_ and Sq_Aβ23_ were high for first-generation binders and in the range of K_D_ 10–15 nM.

### 2.6. Comparison of Expression Levels

Earlier studies on dimeric peptide-binding affibody molecules have indicated that the larger protein structure compared to monomeric three-helical affibody molecules tend to have a negative impact on the yield when recombinantly produced in *E. coli*. The sequestrins Sq_Aβ22_ and Sq_Aβ23_ were expressed in fusion to a *C*-terminal his-tag in *E. coli* and purified using Immobilized Metal Affinity Chromatography (IMAC). A previously described dimeric amyloid-beta binding affibody molecule, Z_SYM73_ [10], was included for comparison and the results showed around 2-fold higher yield for the new sequestrins compared to Z_SYM73_ (Table 2).

### 2.7. Analysis of Changes in Secondary Structure Content upon Target Binding

**Figure 5:**
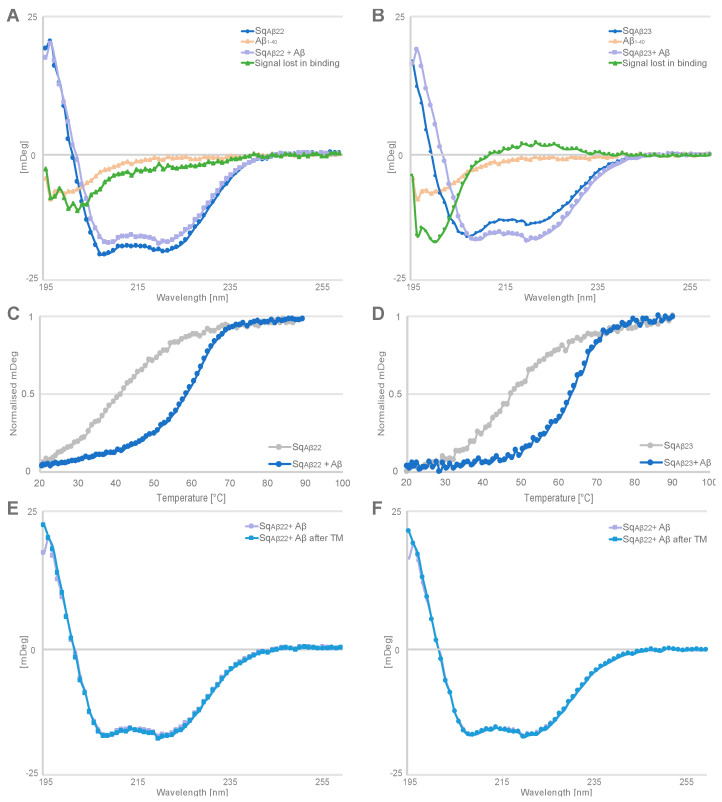
Circular dichroism (CD) spectra between 195–260 nm for proteins in equimolar concentrations of 15.7 μM for (**A**,**B**) Indicates the structural rearrangement upon co-incubation of peptide and sequestrins (light purple). The green line shows the signal lost upon co-incubation between Aβ (beige) and the sequestrin (blue). (**C**,**D**) Variable temperature measurements at 221 nm of Sq_Aβ22_ and Sq_Aβ23_ with and without Aβ co-incubated with the sequestrins. (**E**,**F**) Sq_Aβ22_ and Sq_Aβ23_ in complex with Aβ before (light purple) and after (blue) thermal melting.

It has previously been demonstrated that similar homodimeric amyloid-beta binding affibody molecules undergo structural rearrangement upon peptide sequestration, forming a four-stranded beta-sheet comprised of one strand from each subunit and a beta hairpin from the amyloid-beta peptide [7]. CD spectroscopy was used to reveal indications of similar structural rearrangements in Sq_Aβ22_ and Sq_Aβ23_ upon binding Aβ. First, CD spectra for free Aβ_1–40_ peptide and sequestrin were recorded, respectively, and thereafter compared to the spectra for co-incubated Aβ_1–40_ peptide and sequestrin. The sum of the ellipticities for the free interactants were then subtracted from the ellipticities recorded for the co-incubated samples, demonstrating a change in signal thus indicating a change in secondary structure content upon binding (Figure 5A,B). The secondary structure content was approximated using the BeStSel algorithm [19], revealing a loss of unstructured peptide content, a decrease in alpha helicity and gain of beta sheet content for the samples co-incubated with Aβ peptide and sequestrin, and the effect was more prominent for Sq_Aβ23_. Next, the melting temperatures for the co-incubated samples were determined, demonstrating that binding to the Aβ peptide resulted in a substantial increase in T_m_, from 42 to 59 °C for Sq_Aβ22_ and 48 to 68 °C for Sq_Aβ23_ (Figure 5C,D). In a control experiment, the CD spectrum for Aβ_1–40_ peptide was recorded, as well as the ellipticity for Aβ_1–40_ peptide at 221 nm in a variable temperature measurement, showing negligible and relatively stable signal at 221 nm in that temperature range (Figure S5).

Finally, as for free the free sequestrins, Sq_Aβ22_ and Sq_Aβ23_ co-incubated with Aβ_1–40_ peptide demonstrated full refolding after heat-induced denaturation (Figure 5E,F).

### 2.8. Aβ Aggregation

**Figure 6:**
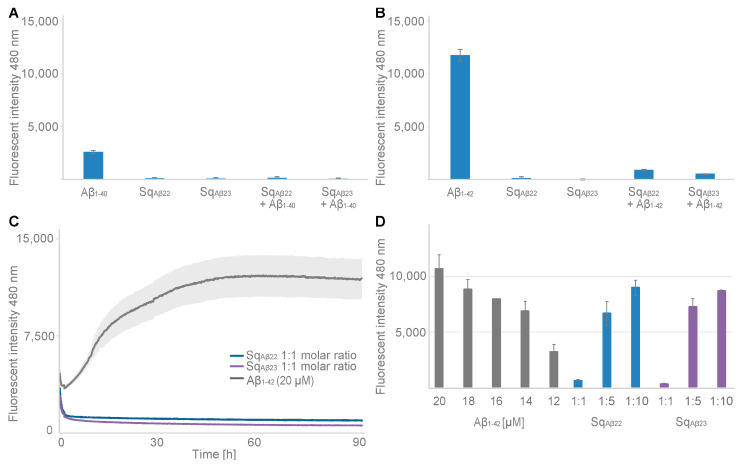
Aggregation assay measuring thioflavin T (ThT) fluorescence at 480 nm. (**A**) Histogram showing endpoint ThT fluorescence at 92 h for 20 μM Aβ_1–40_, 20 μM Sq_Aβ22_, 20 μM Sq_Aβ23_, 1:1 molar ratio of 20 μM Aβ_1–40_ + 20 μM Sq_Aβ22_ and 1:1 molar ratio of 20 μM Aβ_1–40_ + 20 μM Sq_Aβ23_. (**B**) Histogram showing endpoint ThT fluorescence at 92 h for 20 μM Aβ_1–42_, 20 μM Sq_Aβ22_, 20 μM Sq_Aβ23_, 1:1 20 μM Aβ_1–42_ + 20 μM Sq_Aβ22_ and 1:1 20 μM Aβ_1–42_ + 20 μM Sq_Aβ23_. (**C**) ThT fluorescence monitored for 92 h for 20 μM Aβ_1–42_ (grey), 1:1 molar ratio of 20 μM Aβ_1–40_ + 20 μM Sq_Aβ22_ (blue) and 1:1 molar ratio of 20 μM Aβ_1–40_ + 20 μM Sq_Aβ23_ (purple). (**D**) Histogram showing endpoint ThT fluorescence at 92 h for 12–20 μM Aβ_1–40_ (in grey), and sequestrins Sq_Aβ22_ (blue) and Sq_Aβ23_ (purple) at different molar ratios of 1:1, 1:5 and 1:10 in 20 μM Aβ_1–42_. Standard deviation of the replicates is shown in the graph.

Potential inhibition of amyloid-beta aggregation was assessed using a thioflavin T (ThT)-based fluorescence-spectroscopy assay. ThT fluorescence, corresponding to Aβ peptide aggregation, was monitored for 92 h in a fluorescence microplate reader. In addition to Aβ_1–40_, the more aggregation-prone Aβ_1–42_ was also included in the analysis. First, ThT fluorescence was measured at 92 h for 20 μM Aβ_1–40_ and Aβ_1–42_, respectively, showing the expected aggregation, and higher signal for Aβ_1–42_ (Figure 6A,B). As a control, the sequestrins Sq_Aβ22_ and Sq_Aβ23_ were incubated with ThT fluorophore and the fluorescence was recorded for 92 h, demonstrating negligible signal compared to the Aβ peptides (Figure 6A,B). Next, Aβ_1–40_ and Aβ_1–42_, respectively, were co-incubated with equimolar concentrations (1:1) of respective sequestrin, showing very low signals comparable to background for all four samples (Figure 6A,C). Moreover, ThT fluorescence was monitored for five different concentrations of Aβ_1–42_, ranging from 12 to 20 μM, showing the expected aggregation and a concentration-dependent maximum signal (Figure 6D). The sequestrins are expected to bind to monomeric Aβ peptide with 1:1 stoichiometry. Nevertheless, the assay was also conducted with lower sequestrin-to-peptide ratios, corresponding to 4 μM sequestrin in 20 μM Aβ peptide (i.e., 1:5 ratio), as well as 2 μM sequestrin in 20 μM Aβ peptide (i.e., 1:10 ratio). The results showed lower and incomplete inhibition of the aggregation, with signals at 92 h that were similar to the signals corresponding to 16 μM free Aβ peptide for the 1:5 ratio and 18 μM free Aβ peptide for the 1:10 ratio (Figure S6).

## 3. Discussion

Neurodegenerative disorders are a huge burden for society and the burden is expected to grow with the increasing global life expectancy [20]. A better understanding of the molecular mechanisms behind such diseases and finding suitable diagnostics and treatment strategies is thus essential.

In an effort to contribute with new molecular tools for studies and inhibition of peptide aggregation in neurodegenerative disorders, a new protein scaffold was designed, intended for generation of affinity reagents for peptides such as the amyloid beta (Aβ) peptide.

Based on data from previous research on related affibody-based peptide binders, a head-to-tail dimeric protein scaffold (denoted sequestrin) was designed and used for construction of a combinatorial protein library, containing around 5 × 10^9^ variants. The sequestrin library was displayed on phage and used in biopanning against the Aβ peptide to assess the utility of the scaffold and the library design in respect of development of peptide-binding affinity reagents. The library design resulted in a theoretical diversity of 1.27 × 10^22^ variants. The obtained phage library, containing 5 × 10^9^ individual clones, is thus only covering a very small fraction of all possible combinations. However, the library was intentionally designed to include many randomized positions in the protein, given the limited amount of information on this new type of protein scaffold and mode of binding. The long-term aim is that selections, followed by sequencing and characterization of binders will result in knowledge that will guide the design of improved next-generation sequestrin libraries in the future. Moreover, the recent substantial improvement in computational methods for predicting protein folding and protein–protein interactions, such as neural network-based models [21], paves the way for computer-aided library design based on deep-sequencing data on naïve, displayed and enriched library clones in the future.

A tendency of growth bias was observed during amplification of the phagemid library, which is likely due to the more complex protein structure compared to monomeric affibody molecules. Adjusting the amplification conditions during cultivation as described above was critical for ensuring a successful selection procedure.

Sequencing of the naïve library revealed that the average number of mutations per sequestrin was around seven, which after selection had shifted to around four among the enriched peptide-binding variants. Future selections against other targets will show if this trend continuous and could hopefully be valuable for further developments of the sequestrin scaffold for protein engineering.

Based on the most reoccurring sequences and taking into account representation from the different selection cycles (3, 4, and 5), 13 clones were selected for characterisation. The thirteen sequestrins were produced, purified, and initially analysed in terms of secondary structure content, thermal stability, refolding and affinity.

Eleven of the thirteen candidates showed high affinity for the amyloid-beta peptide with K_D_ in the 1–30 nM range, characterized by relatively slow kinetics for both the association and the dissociation. The variants with highest affinity (Sq_Aβ22_ and Sq_Aβ23_) originated from the fifth selection cycle, indicating that the increased stringency in the later cycles indeed enriched for high-affinity binding. As expected, analysis of the interaction at 37 °C demonstrated faster dissociation, corresponding to around 5- to 10-fold higher K_D_. It should be noted that immobilizing the Aβ peptide on the chip probably results in an underestimation of the affinity. Previously reported SPR-based studies on structurally related dimeric affibody molecules for Aβ revealed a relatively large difference when the affinity was determined using SPR compared with analysis in solution [10]. This is likely due to the structural rearrangements of both interactants upon binding [7], which might be slower when the Aβ peptide is immobilized on the chip surface.

To get an indication whether similar structural rearrangement also occurs when the new sequestrins bind to the Aβ peptide, circular dichroism (CD) spectroscopy was used to detect potential changes in secondary structure content upon binding. The analysis showed that the Aβ peptide is unstructured in solution and the secondary structure content of the sequestrins is largely alpha helical. Co-incubation of Aβ peptide and respective sequestrin resulted in changes in the CD spectrum compared with the sum of CD spectra of the free interactants. Moreover, the thermal stability of the complex was increased 17–20 °C compared to the T_m_ for the free sequestrins. In future studies, determining the three-dimensional structure of the complex would confirm whether these results are indeed due to structure rearrangements in the sequestrins and the Aβ peptide, similar to what has been observed for dimeric peptide-binding affibody molecules [7].

Finally, the effect from binding on the aggregation propensity of Aβ was assessed using a ThT fluorescence assay. The results showed that both sequestrins fully inhibited aggregation of Aβ when added at a 1:1 molar ratio, both for Aβ_1–40_ and for the more aggregation-prone variant Aβ_1–42_. Future studies on the effects of the new sequestrins on Aβ pathology in AD models is hence warranted. A challenge when targeting neurodegenerative diseases with biologics is the limited uptake across the blood-brain-barrier. Hence, genetic fusion of the sequestrins with a domain for transferrin receptor (TfR)-mediated transcytosis transportation [22] would be interesting.

In summary, the results show that the design of the new sequestrin scaffold and library is suitable for selection of high-affinity peptide binders using phage display technology. Future selections against other aggregation-prone peptides, such as alpha synuclein, tau and TDP-43 will hopefully further validity the utility of this new class of affinity reagent.

## 4. Materials and Methods

### 4.1. Library Design and Molecular Cloning

Two randomized double stranded DNA oligos, encoding each of the two sequestrin (Sq) subunits, were purchased from TWIST Bioscience (San Francisco, CA, USA) for assembly into one library oligo by hybridization of overlapping bases in each subunit: 5′-GCGGGTGGCGAANNNNNNNNNNNNCCGAACTTANNNNNNGACCAANNN-TGTGCCNNNNNNCGTAGTNNNGAAGATGATCCTAGTCAAAGCGCTAACTTG-NNNGCAGAAGCTAAAAAGCTAAATGATGCTCAGGCGCCGGCGAGCAGCAGCAGCGGGAGCAGCAGCAGCGGGCGCGCGAGTGCGGGTGGCGAGNNNNNN-NNNNNNCCGAACTTANNNNNNGACCAANNNTGTGCCNNNNNNCGTAGTNNNGAGGATGACCCTAGTCAAAGCGCTAACTTGNNNGCAGAAGCTAAAAAGCT-AAATGATGCTCAGGCGCCGAAA -3′ (with randomized codons illustrated as NNN). The genes were flanked by BamHI and SalI restriction sites for subcloning in fusion to a gene for an albumin-binding domain (ABD; [17]) into the pAffi1 phagemid [5]. Each subunit of the library was amplified by polymerase chain reaction (PCR) in 12 cycles using Phusion DNA polymerase (New England Biolabs, Ipswich, MA, USA) and primers introducing 60 overlapping bases into the two oligo subunits for subsequent hybridization. The PCR products were purified using a PCR purification kit (Qiagen GmbH, Hilden, Germany). Equimolar amounts of the oligo subunits were hybridized into one long library gene of 321 bp, which was subsequently PCR-amplified in 10 cycles using Phusion DNA polymerase (New England Biolabs, Ipswich, MA, USA). PCR products of correct length were purified by preparative gel electrophoresis (2% agarose gel) followed by purification using a QIAquick gel purification kit (Qiagen GmbH, Nordrhein-Westfalen, Germany). Purified PCR products were digested by BamHI and SalI (New England Biolabs, Ipswich, MA, USA) enzymes. The modified pAffi1 vector was digested by the same enzymes and purified by preparative gel electrophoresis. Purified sequestrin fragments were ligated to the phagemid vector as *N*-terminal fusion to a gene encoding an albumin-binding domain (ABD) [17], using T4 DNA ligase (New England Biolabs, Ipswich, MA, USA) at a 1:6 molar ratio of vector to insert. Ligated vector was purified using a QIAquick PCR purification kit (Qiagen GmbH, Germany) before transformed into TG1 electrocompetent cells (Lucigen, Middleton, WI, USA).

Phage stocks were created by standard procedures in XL1Blue cells (Aglient, Santa Clara County, CA, USA). Briefly, superinfection was performed using 5-fold excess of M13K07 phage (New England Biolabs, Ipswich, MA, USA) and precipitation of phage particles was performed using polyethylene glycol (PEG)/NaCl to yield phage titres of approximately 10^13^ pfu/mL.

### 4.2. Library Validation

Superinfected bacterial colonies were individually screened for (1) library size by titration, (2) insert length by PCR amplification using DreamTaq DNA Polymerase (Thermo Scientific, Waltham, MA, USA) and gel electrophoreses, and (3) DNA sequence by Sanger sequencing (Microsynth SeqLab, Gottingen, Germany). Monoclonal phage ELISA was used to validate expression of recombinant protein on the phage surface by ABD binding to human serum albumin (HSA). Individual clones were cultivated in 96-deep well format in tryptic soy broth medium supplemented with yeast extract (TSBY) and 100 μg/mL carbenicillin at 30 °C, 250 rpm, ON. After ON, cultures were re-inoculated and incubated at 37 °C, 250 rpm until OD_600_ reached 0.5–0.8 AU. Superinfection with 0.3 × 10^6^ pfu/clone M13K07 (New England Biolabs, Ipswich, MA, USA) at 37 °C without rotation for 30 min proceeded induction with 1 mM Isopropyl β-D-1-thiogalactopyranoside (IPTG; Chemtronica, Stockholm, Sweden) and further cultivation ON at 37 °C, 250 rpm, under additional antibiotic pressure from 30 μg/mL kanamycin. Harvested phage supernatants were incubated for 1 h in 384-microwell plates (Nunc, PS, Low binding, Hi-Edge, clear) precoated with HSA [5 μg/mL] or bovine serum albumin (BSA) [1 *w*/*v*%]. The ELISA plate was blocked with 1 *w/v*% BSA prior to phage incubation to minimize background, and plates were washed in phosphate-buffered saline + 0.05% Tween-20 (PBST) before incubating with a mouse monoclonal M13 Bacteriophage Antibody (HRP) (Sino biological, Beijing, China) according to supplier’s instructions. Signal development was done with Pierce™ TMB Substrate Kit (Thermo Scientific, Waltham, MA, USA), as by instructions. After colorimetric development, the reaction was terminated by addition of 2 M H_2_SO_4_. Absorbance was measured at 450 nm using a CLARIOStar Plus plate reader (BMG Labtech, Ortenberg, Germany). DNA sequencing was performed by Sanger sequencing (Microsynth SeqLab, Göttingen, Germany), and sequences analysed with the Geneious software (version 11.2, Biomatters LTD, Auckland, New Zealand).

### 4.3. Selections of Sequestrins against Amyloid Beta

The sequestrin library phage stock (denoted Sq_lib_) was used in selections against *C*-terminally biotinylated Aβ_1–40_ peptide (AnaSpec, Fremont, CA, USA) in a total of five rounds with decreasing amount of soluble target antigen in each round (50 nM, 40 nM, 20 nM, 10 nM, 1 nM). The incubation temperature varied between the cycles (4 °C ON for cycle 1, 1 h RT for cycle 2–4, 1 h 45 °C for cycle 5) before antigen was captured by Dynabeads M-280 Streptavidin beads (Invitrogen, Waltham, MA, USA). All cycles were preceded with a negative selection round towards beads pre-blocked with 5 *w*/*v*% BSA. The stringency of PBSTB (0.1% Tween-20, 3 *w*/*v*% BSA) washes was increased with each selection round (2 × 1 min, 4 × 1 min, 5 × 3 min, 5 × 6 min, 4 × 6 min + 1 × 2 h + 1 × 6 min), where the last wash was done in PBS buffer. Phage eluates were obtained by incubation for 10 min in 0.1 M glycine-HCl, pH 3.0, followed by neutralization by Tris-HCl, 1 M, pH 8.0. Eluates were amplified by infection with 100× excess of XL1Blue *E. coli* cells, followed by plating on Aquare BioAssay Dish (Corning, Somerville, MA, USA) with blood agar base (BAB; Merck, Darmstadt, Germany) supplemented with 2% D-glucose, 100 μg/mL carbenicillin, and 10 μg/mL tetracycline and incubated at 37 °C ON. Bacterial colonies were recovered by addition of TSBY medium to the plates, followed by scraping off colonies from the plates and dissolving the cells in TSBY before continuing cultivation in a suspended format. The cultures containing infected bacteria with the eluted phage (>100× excess compared to eluate complexity) were reinoculated to OD_600_ = 0.2 AU, followed by superinfection with five times excess of M13K07 phage at an OD_600_ = 0.8 AU.

### 4.4. Phage ELISA

Amplified phage stock and individual clones after each selection round were cultured for polyclonal and monoclonal phage ELISA, respectively, as described above. All phage stocks were diluted to the same concentration before incubating with target antigen.

ELISA plates in 384-well format (Nunc, PS, Low binding, Hi-Edge, clear) were prepared with four wells per individual sample according to: (1) one well coated with 5 μg/mL HSA, (2) one well coated with 1 *w*/*v*% BSA, (3) one well pre-coated with 5 μg/mL streptavidin followed by 1 μg/mL biotinylated amyloid beta peptide (Aβ_1–40_; AnaSpec, USA) and (4) one well pre-coated with 5 μg/mL streptavidin followed by 1 *w*/*v*% BSA. Development and washes were performed as described above. The signal, representing background, from wells with BSA (for 1) or streptavidin-BSA (for 3) were subtracted. The signal from wells with Aβ_1–40_ was normalized with the signal from wells with HSA. DNA sequences were identified by Sanger sequencing (Microsynth SeqLab, Göttingen, Germany), and the sequences were analysed with the Geneious software (version 11.2, Biomatters LTD, Auckland, New Zealand). 

### 4.5. Expression and Purification of Soluble Sequestrins

Sequences from the selections were chosen for further characterization. Sequestrin-encoding DNA was amplified from phagemids with primers designed for the In-Fusion HD cloning kit (Takara Bio Europe, Göteborg, Sweden), according to manufacturer’s recommendations into the pET-26b(+)-vector for periplasmic production with a *C*-terminal His_6_-tag. Sequence-verified clones (Sanger sequencing, Eurofins Genomics GmbH, Ebersberg, Germany) were transformed using heat shock to *E. coli* BL21(DE3) for recombinant protein expression. Cultures were inoculated to an OD_600_ of 0.1 AU and incubated in TSBY with 25 μg/mL kanamycin at 37 °C until OD_600_ reached 1.0 AU before inducing with 1 mM IPTG and thereafter incubated at 25 °C for 16 h. Cell pellets from harvest were dissolved in running buffer [47 mM Na_2_HPO_4_, 3 mM NaH_2_PO_4_, 300 mM NaCl, 15 mM imidazole, pH 7.4] before proceeding with purification. Cells were sonicated using a Vibra-Cell VCX 130 sonicator (Sonics, Newtown, CT, USA) and cell debris was removed by centrifugation and filtration. Cell lysate was loaded on an equilibrated HisPur Cobalt Resin (Thermo Scientific, Waltham, MA, USA) and washed with running buffer. The sample was eluted with running buffer supplemented with 150 mM imidazole. Fractions of eluate containing protein according to analysis with the Pierce™ BCA Protein Assay Kit (Thermo Scientific, Waltham, MA, USA), performed according to manufacturer’s instructions, were pooled and buffer exchanged to PBS on PD 10 desalting columns (Cytiva, Marlborough, MA, USA). The samples were analysed with Sodium Dodecyl Sulfate–PolyAcrylamide Gel (SDS-PAGE) (NuPAGE Bis-Tris 4–12%, Invitrogen, Waltham, MA, USA). Molecular mass was analysed by electrospray ionization mass spectrometry on a Thermo Ultimate3000 Bruker Impact II system connected to a ProSwift RP-4H, 1 × 50 mm column (Thermo Fisher, Waltham, MA, USA) using a linear gradient elution with acetonitrile (3 to 95%), supplemented with 0.1% formic acid. The instrument was run with settings for electrospray ionization with a positive ion polarity, spanning the mass range 500 to 50,000 *m*/*z*.

### 4.6. Circular Dichroism Spectroscopy

The secondary structure content of the sequestrins was analysed using circular dichroism spectroscopy on a Chirascan system (Applied Photophysics, Leatherhead, UK) with a 1 mm High precision cell (110-1P-40 cuvettes, Hellma Analytics, Munich, Germany). Five wavelength scans were recorded and averaged between 195 nm and 260 nm at 20 °C on 0.2 mg/mL protein in PBS. Melting point was determined by using a temperature gradient of 1 °C per minute at 221 nm for five average readings. The refolding capability was assessed by repeating the spectral scan after the sample had been subjected to heat treatment and cooled down to 20 °C. The spectra from before and after heating was compared to assess refolding. Respective sequestrin and Aβ_1–40_ (AnaSpec, USA) were co-incubated at equimolar concentrations of 15.7 μM and analysed by circular dichroism for analysis of changes in secondary structure content upon interaction. Secondary structure content was approximated by BeStSel algorithm [19].

### 4.7. Surface Plasmon Resonance Assay for Analysis of Binding to Aβ_1–40_

Surface plasmon resonance (SPR) on a Biacore 8K instrument (Cytiva, Marlborough, MA, USA) was used to determine affinity and kinetics of sequestrins binding to Aβ_1–40_. Series S SA sensor chips (Cytiva, Marlborough, MA, USA) were immobilized with 120 response units (RU) of biotinylated Aβ_1–40_ (AnaSpec, Fremont, CA, USA) according to manufacturer’s instructions. PBST (0.05% Tween-20) was used as running buffer. All protein candidates were injected as a multi-cycle analysis in an 8-step 1:1.5 dilution series from 342 nM to 30 nM at 25 °C in duplicate. The dimeric affibody Ztaq_3638_-(G_4_S)_2_-Ztaq_3638_-ABD was used as negative control [18]. Analyte was injected for 200 s and dissociation was recorded for 600 s at 30 μL/min. Surfaces were regenerated with 10 mM HCl for 35 s and let to stabilize for 30 s before next cycle. The results were evaluated using the Multi-cycle kinetics method—1:1 binding, using the Biacore Insight Evaluation Software (Version 2.0.15, Cytiva, Marlborough, MA, USA).

In a second analysis, Series S SA sensor chips were immobilized with 80 response units (RU) of biotinylated Aβ_1–40_, and Sq_Aβ22_, and Sq_Aβ23_ were injected as analyte in a 1:2 dilution series ranging from 300 nM to 75 nM at 37 °C in duplicate.

### 4.8. Aggregation Assay

Fibrillization inhibition assays were done for the Aβ_1–40_ (#AS-24236, AnaSpec, Fremont, CA, USA), and Aβ_1–42_ (#AS24224, AnaSpec, Fremont, CA, USA) using the sequestrins Sq_Aβ22_ and Sq_Aβ23_ as inhibitors for aggregation at decreasing molar ratios 1:1, 1:5, and 1:10 in relation to the 20 μM of Aβ. All proteins were thawed on ice and spun down at 10,000× *g* for 5 min at 4 °C to remove precipitates. Each sample was prepared in triplicate in a volume of 60 μL per reaction by adding PBS (Aβ_1–40_: 10 mM phosphate and 150 mM NaCl, pH 7.2; Aβ_1–42_: 10 mM phosphate and 150 mM NaCl, pH 8.0), 20 μM Thioflavin T (ThT) dye, and 20 μM sequestrin before vortexing for 3 s and kept on ice. When all samples were prepared the Aβ peptide was added at 20 μM concentration to all sequestrin samples, or as a standard at 12–20 μM, to the tubes and vortexed for 1 s. The sample was added to a 384-well plate (Nunc, PS, Low binding, Hi-Edge, clear) with surrounding wells filled with PBS, and sealed to minimize evaporation during analysis. The fluorescence intensity was immediately analysed in a CLARIOStar Plus (BMG Labtech, Ortenberg, Germany) with excitation at 440–10 nm and emission at 480–10 nm. The readings were done during 999 cycles with a cycle time of 325 s at 37 °C with 50 flashes with 0.1 s settling time per well, using 1500 gain adjustment. The plate was shaken in an orbital motion 500 rpm for 1 s before each measurement. Analysis was done in the Mars analysis software (version 3.0, BMG Labtech, Ortenberg, Germany) where replicates were averaged, and blank subtracted from well containing only PBS with ThT dye.

## Figures and Tables

**Table 1:** Affinity data from SPR data fittings, thermal melting points (T_m_) as well as refolding capabilities as assessed by CD.

Analyte	k_a_ [1/Ms]	k_d_ [1/s]	K_D_ [nM]	T_m_ [°C]	Refolding
Sq_Aβ1_	1.9 × 10^4^	1.71 × 10^−4^	8.40	42	Yes
Sq_Aβ2_	2.97 × 10^4^	4.24 × 10^−4^	14.3	37	Yes
Sq_Aβ6_	1.55 × 10^4^	3.16 × 10^−4^	20.4	46	Yes
Sq_Aβ7_	9.21 × 10^3^	2.12 × 10^−4^	23.1	42	Yes
Sq_Aβ8_	n.d.	n.d.	n.d.	n.d.	Partly
Sq_Aβ9_	2.48 × 10^4^	9.49 × 10^−4^	38.3	46	Partly
Sq_Aβ10_	5.13 × 10^4^	2.62 × 10^−4^	5.12	37	Partly
Sq_Aβ14_	1.56 × 10^4^	4.47 × 10^−4^	28.6	35	Yes
Sq_Aβ15_	7.52 × 10^3^	9.04 × 10^−4^	121	38	Yes
Sq_Aβ16_	4.98 × 10^4^	5.65 × 10^−4^	11.4	41	Yes
Sq_Aβ20_	2.12 × 10^4^	2.13 × 10^−4^	10.0	47	Yes
Sq_Aβ22_	1.63 × 10^4^	1.70 × 10^−5^	1.13	42	Yes
Sq_Aβ23_	2.16 × 10^4^	7.05 × 10^−5^	3.36	48	Yes

**Table 2:** Production yields compared to Z_SYM73_. Expected mass in molecular weight (Mw)and observed molecular mass as determined by Mass spectrometry (MS).

Construct Zseqlib Clone	Yield Protein per 100 mL Culture [mg]	Fold Improved Expression Compared to Z_SYM73_-His_6_	Mw Expected [Da]	Mw Observed [Da]
Z_SYM73_-His_6_	2.8+/−1.9	n.a.	12,331	12,329
Sq_Aβ22_-His_6_	6.7+/−1.4	2.4	12,528	12,527
Sq_Aβ23_-His_6_	6.1+/−1.3	2.2	12,607	12,605

## Data Availability

Not applicable.

## Supplementary Materials

The following supporting information can be downloaded at: https://www.mdpi.com/article/10.3390/ijms24010836/s1, Figure S1: Phage display selection against Aβ; Figure S2: Sequence distribution of sequestrins. Table S1: Sequence of analysed sequestrins. Table S2: Molecular mass of produced sequestrins. Figure S3: Circular dichroism of produced sequestrins. Figure S4: SPR sensorgrams of sequestrins against Aβ peptide. Figure S5: Circular dichroism of Aβ peptide. Figure S6: Aggregation inhibition of Aβ peptides.
