# Supplementary material for: Construction and Validation of a New Naïve Sequestrin Library for Directed Evolution of Binders against Aggregation-Prone Peptides
Source: Int J Mol Sci. 2023 Jan 3;24(1):836. doi: 10.3390/ijms24010836 (PMC9821733; doi:10.3390/ijms24010836)
Supplement: Supplementary file 1 [file ijms-24-00836-s001.zip › ijms-2134571-supplementary.pdf]

Supplementary material

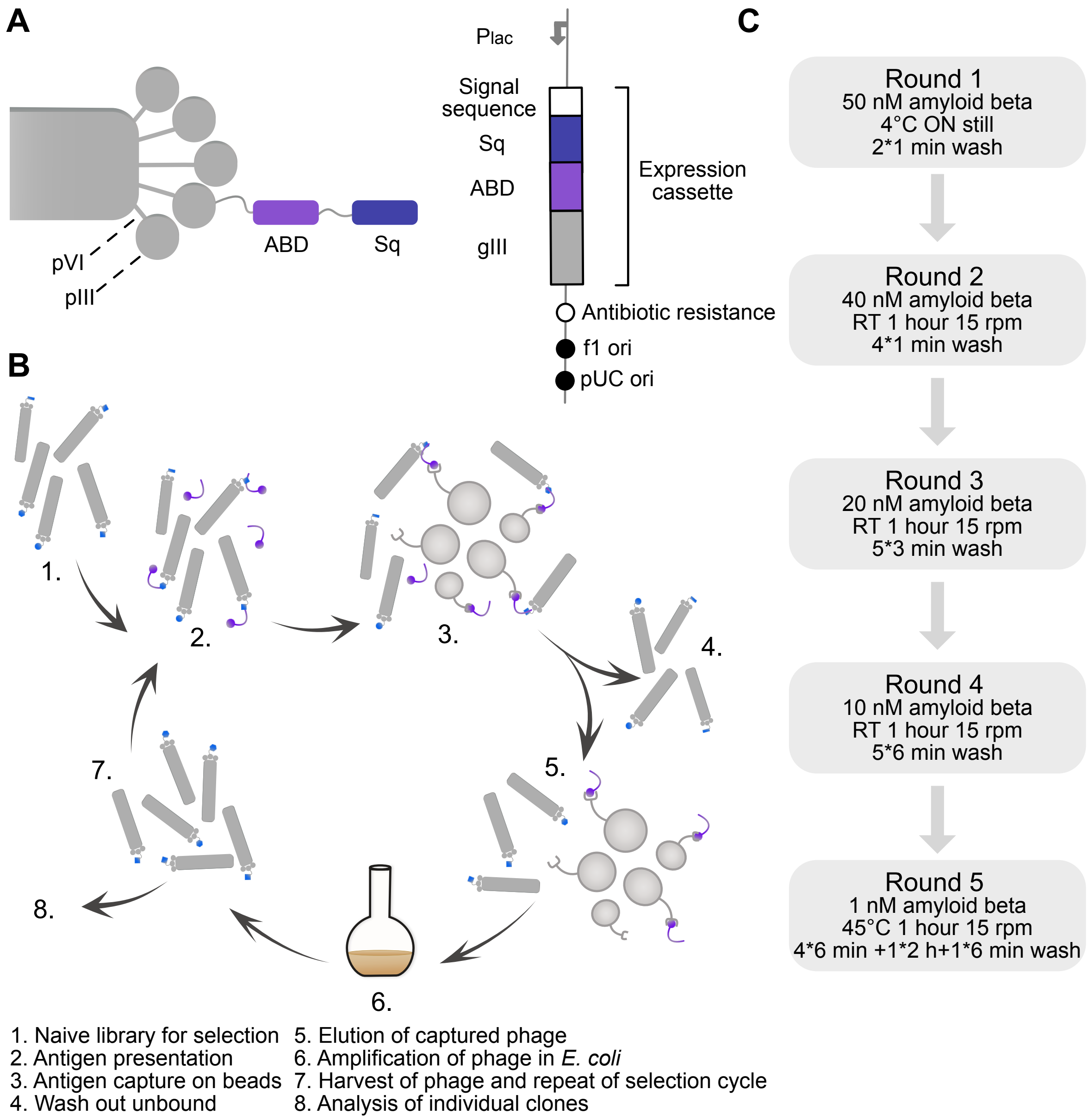

Figure S1. (A) *Phagemid vector and display of sequestrin (Sq) in fusion to albumin binding domain (ABD) on phage.* ***(B)*** *Schematic illustration of phage display selection with magnetic beads for capture of biotinylated target antigen, repeated for the number of desired cycles.* ***(C)*** *Design of selection cycles against Aβ with the sequestrin library.*

A

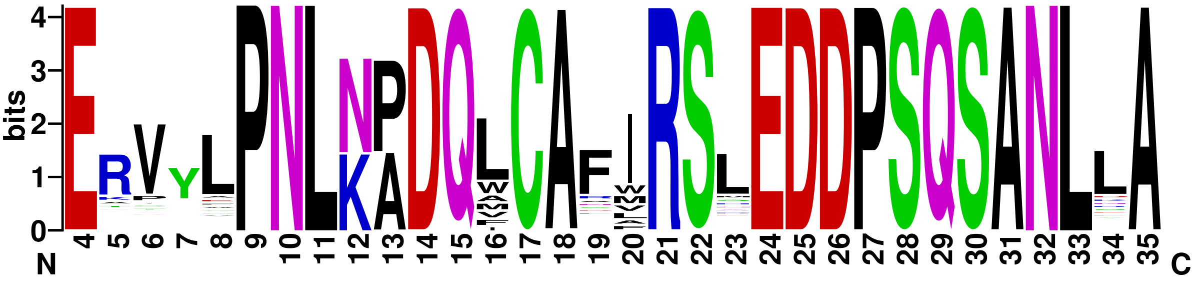

B

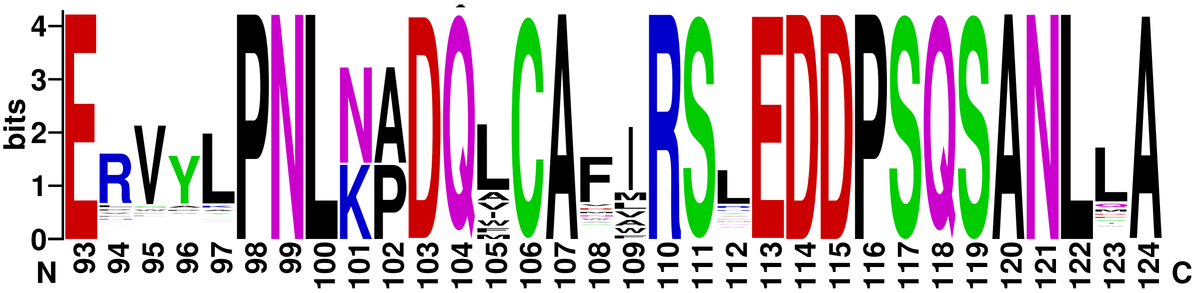

C

	Naive library Position 5	Selected Position 5	Naive library Position 6	Selected Position 6	Naive library Position 7	Selected Position 7	Naive library Position 8	Selected Position 8	Naive library Position 12	Selected Position 12	Naive library Position 13	Selected Position 13	Naive library Position 16	Selected Position 16	Naive library Position 19	Selected Position 19	Naive library Position 20	Selected Position 20	Naive library Position 23	Selected Position 23	Naive library Position 34	Selected Position 34
Unknown X	0	0	0	0	0	0	0	0	0	0	0	0	0	0	0	0	0	0	0	0	0	0
H	3	6	1	3	3	0	2	0	0	0	0	0	0	0	1	0	0	0	4	0	1	0
R	54	72	1	0	1	0	1	0	0	0	0	0	0	0	6	0	0	0	4	0	4	0
K	7	8	0	0	3	0	1	0	45	47	0	0	0	0	1	0	0	0	0	0	4	3
E	1	0	2	0	4	0	4	0	0	0	0	0	0	0	2	0	0	0	3	0	3	0
D	3	0	2	0	1	0	1	0	0	0	0	0	0	0	3	0	0	0	2	0	4	0
Q	2	0	1	0	3	0	3	0	0	0	0	0	0	0	2	0	0	0	4	0	3	0
N	1	0	1	0	3	0	1	0	55	53	0	0	0	0	4	0	0	0	4	0	4	0
Y	1	0	1	0	51	86	0	0	0	0	0	0	0	0	2	0	0	0	1	3	3	0
C	0	0	0	0	0	0	0	0	0	0	0	0	0	0	0	0	0	0	0	0	0	0
T	5	0	3	0	3	0	0	0	0	0	0	0	0	0	2	0	0	0	3	3	2	3
S	3	3	3	0	2	0	0	0	0	0	0	0	0	0	4	0	0	0	5	0	4	0
P	2	8	6	0	3	0	2	0	0	0	54	28	0	0	0	0	0	0	0	0	0	0
W	3	0	2	0	3	6	3	0	0	0	0	0	12	3	1	0	9	0	2	0	0	0
F	1	0	0	0	2	6	3	3	0	0	0	0	7	0	56	97	5	3	3	0	3	0
M	2	0	1	0	1	0	1	0	0	0	0	0	7	0	2	0	8	17	5	6	2	6
I	2	0	3	6	4	3	2	0	0	0	0	0	5	3	3	0	60	67	3	3	2	0
L	0	0	2	3	3	0	63	97	0	0	0	0	56	92	3	0	6	0	53	81	55	86
V	2	3	66	89	4	0	3	0	0	0	0	0	7	3	3	3	7	8	2	3	1	3
A	5	0	2	0	4	0	4	0	0	0	46	72	7	0	4	0	5	6	1	3	2	0
G	3	0	2	0	2	0	3	0	0	0	0	0	0	0	0	0	0	0	0	0	0	0

D

	Naive library Position 5	Selected Position 5	Naive library Position 6	Selected Position 6	Naive library Position 7	Selected Position 7	Naive library Position 8	Selected Position 8	Naive library Position 12	Selected Position 12	Naive library Position 13	Selected Position 13	Naive library Position 16	Selected Position 16	Naive library Position 19	Selected Position 19	Naive library Position 20	Selected Position 20	Naive library Position 23	Selected Position 23	Naive library Position 34	Selected Position 34
Unknown X	0	0	0	0	0	0	0	0	0	0	0	0	0	0	0	0	0	0	0	0	0	0
H	3	6	1	0	1	0	1	0	0	0	0	0	0	0	1	0	0	0	4	0	1	0
R	59	92	2	0	2	0	1	0	0	0	0	0	0	3	2	0	0	0	6	3	1	0
K	3	0	2	0	0	0	4	0	44	42	0	0	0	0	2	0	0	0	1	0	1	3
E	1	0	2	0	2	0	2	0	0	0	0	0	0	0	3	3	0	0	4	0	1	0
D	1	0	2	0	2	0	0	0	0	0	0	0	0	0	4	0	0	3	1	0	3	0
Q	1	0	2	0	1	0	2	0	0	0	0	0	0	0	0	0	0	0	4	3	6	0
N	2	3	0	0	3	3	2	0	56	56	0	0	0	0	4	0	0	0	1	0	1	0
Y	2	0	0	0	58	94	3	0	0	0	0	0	0	0	1	0	0	0	4	0	1	0
C	0	0	0	0	0	0	0	0	0	3	0	0	0	0	0	0	0	0	0	0	0	0
T	1	0	2	0	1	0	2	0	0	0	0	0	0	0	3	0	0	0	2	0	2	3
S	2	0	2	0	2	0	0	0	0	0	0	0	0	0	3	0	0	0	3	3	3	0
P	4	0	1	0	4	0	1	3	0	0	54	28	0	0	0	0	0	0	0	0	0	0
W	2	0	3	0	2	0	2	0	0	0	0	0	7	11	4	0	7	0	2	0	3	0
F	3	0	1	3	3	0	2	3	0	0	0	0	6	3	56	69	5	8	3	3	3	0
M	3	0	1	0	1	0	2	3	0	0	0	0	6	3	4	0	9	3	4	3	5	3
I	0	0	2	0	2	0	1	0	0	0	0	0	7	0	2	0	57	72	3	6	3	0
L	5	0	2	3	2	3	67	92	0	0	0	0	58	81	1	25	8	3	50	78	60	89
V	4	0	70	94	3	0	2	0	0	0	0	0	8	0	5	3	8	6	4	3	1	3
A	1	0	2	0	5	0	3	0	0	0	46	72	9	0	3	0	7	6	4	0	2	0
G	1	0	3	0	3	0	2	0	0	0	0	0	0	0	0	0	0	0	0	0	0	0

Figure S2. *Sequence data on naïve and enriched library. Library distribution as sequence logo (weblogo.berkeley.edu) for naïve* Sq_{lib} *in* ***(A)*** *positions 4–35 of first subunit and* ***(B)*** *positions 93–124 of second subunit. Distribution of sequestrin pool after five rounds of selections towards* $A\beta_{1\text{–}40}$ *peptide. Distribution by percentage for each randomised position in the naive library and enriched library for the* ***(C)*** *first subunit and* ***(D)*** *second subunit.*

Table S1. *Mutations in randomized positions differing from the designed library (marked with X) for selected set of sequestrins for subcloning. The first and second subunit are different for each sequestrin and expressed as a head-to-tail construct with a S_4G-linker.*

	1 10 20 30 40 50 60 70 80 90 107
Sq_{lib}	AGGEXXXXPNLXXDQXCAXXRSXEDDPSQSANLXAEAKKLNDAQAPASSSSGSSSSGRASAGGEXXXXPNLXXDQXCAXXRSXEDDPSQSANLXAEAKKLNDAQAPK
$Sq_{A\beta1}$	....K...M...........................K...M..............................
$Sq_{A\beta2}$	....T...P.........F...........................
$Sq_{A\beta6}$	...............F..V......K......MM...........................
$Sq_{A\beta7}$	............P..A...........................
$Sq_{A\beta8}$	....P.R........I...A..Y..K.......M...........................
$Sq_{A\beta9}$	....L.............M...W..KP..I...L...........................
$Sq_{A\beta10}$	...........K.......V...KP.................................
$Sq_{A\beta14}$	...................A..S..TP............F...........................
$Sq_{A\beta15}$	...........K..........A..K......V............................
$Sq_{A\beta16}$	...........K.......V...KP..I...L...........................
$Sq_{A\beta20}$	....P.F............M...
$Sq_{A\beta22}$	..................VA...M.............
$Sq_{A\beta23}$	............P..K......L............................

Table S2. *molecular weight of sequestrins after production with hexa-histidine tag. Expected mass and observed mass in MS are correlating well, taking into consideration the disulphide bond formation between cysteines. Clone 8, 9, and 14 however shows a -71 Da difference from expected and observed.*

**Construct Zseqlib clone**	**$Mw_{expected}$ [Da]**	**$Mw_{observed}$ [Da]**
Z_{SYM73}-His_6	12,331	12,329
$Sq_{A\beta1}$-His_6	12,329	12,329
$Sq_{A\beta2}$-His_6	12,492	12,492
$Sq_{A\beta6}$-His_6	12,290	12,290
$Sq_{A\beta7}$-His_6	12,281	12,281
$Sq_{A\beta8}$-His_6	12,271	12,200
$Sq_{A\beta9}$-His_6	12,351	12,280
$Sq_{A\beta10}$-His_6	12,337	12,337
$Sq_{A\beta14}$-His_6	12,199	12,128
$Sq_{A\beta15}$-His_6	12,538	12,537
$Sq_{A\beta16}$-His_6	12,337	12,337
$Sq_{A\beta20}$-His_6	12,543	12,542
$Sq_{A\beta22}$-His_6	12,528	12,527
$Sq_{A\beta23}$-His_6	12,607	12,605

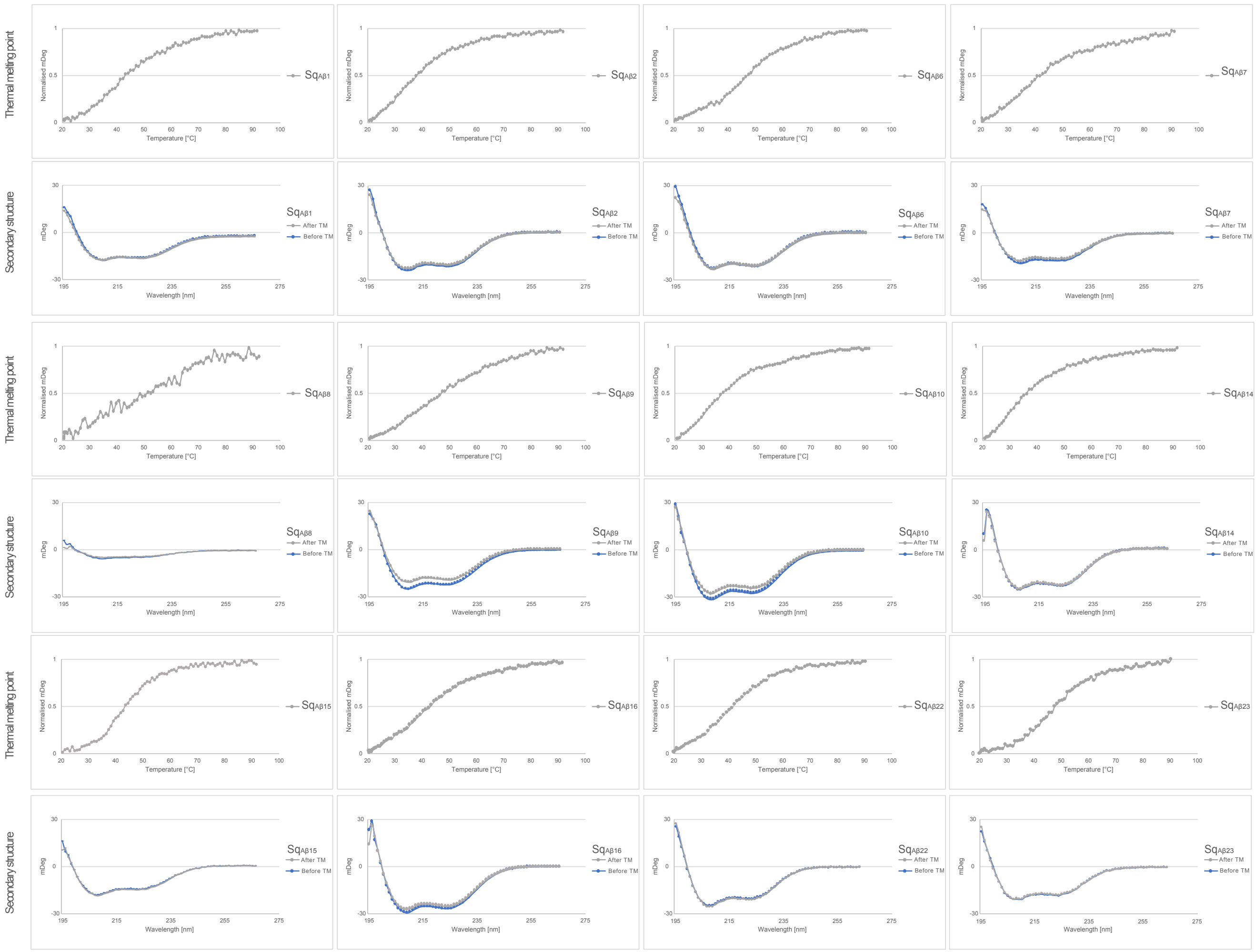

Figure S3. *Circular dichroism (CD) spectroscopy spectra of $Sq_{A\beta}$ clones at 0.2 mg/ml. All clones except $Sq_{A\beta 8}$ show alpha helical secondary structure content structure with two peaks at 221 nm and 208 nm. Most clones show full refolding after heating by complete overlap between spectra before (blue) and after (grey). The concentration of $Sq_{A\beta 8}$ was too low for a reliable determination.*

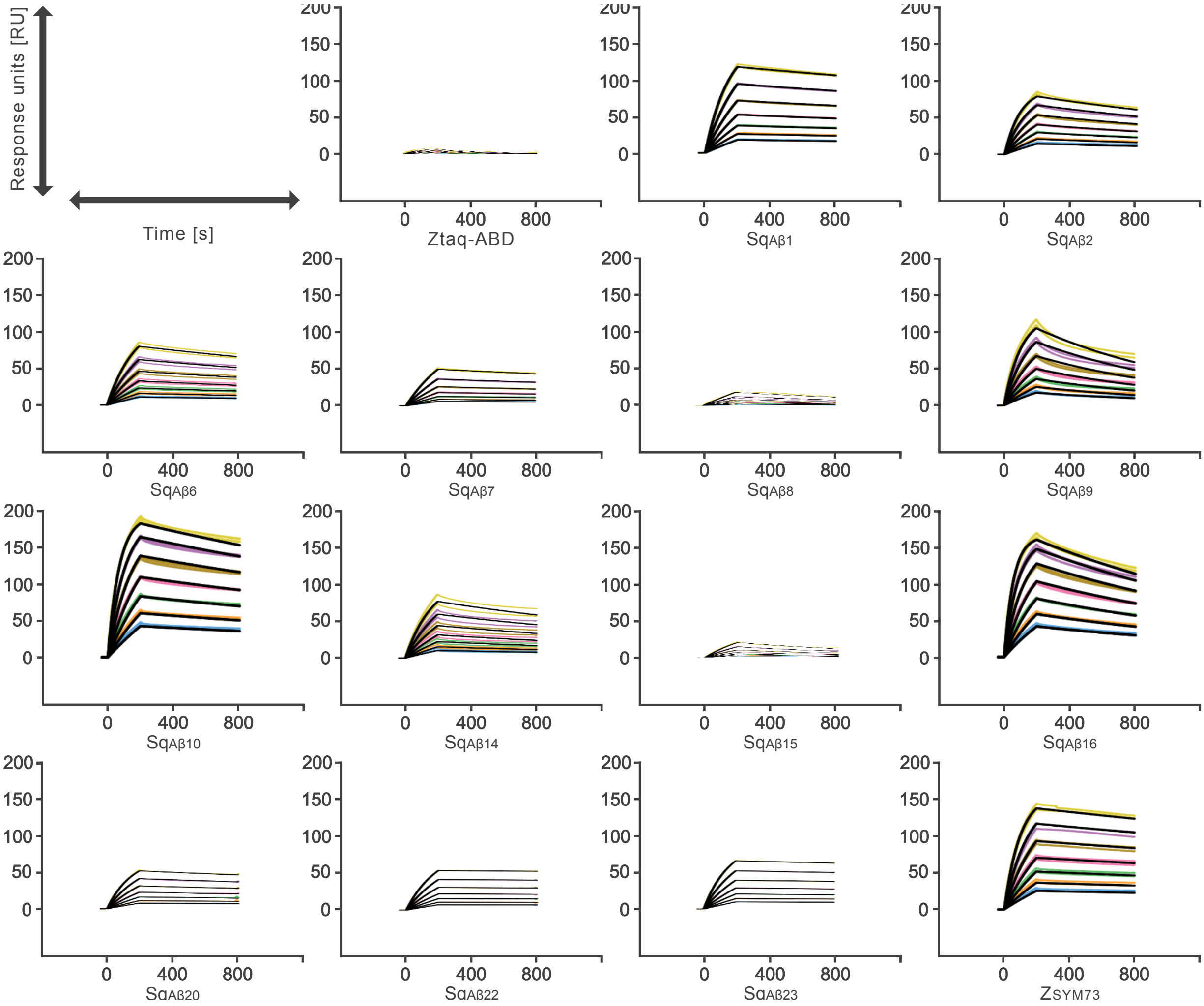

Figure S4. *SPR sensorgrams from dilution series preformed in duplicate for all candidates at 25 °C. X-axis: time in seconds and y-axis: response units (RU). Coloured curves are duplicate injections in the concentration series (1:1.5 steps from 342 to 30 nM). Black curves are from fitting the data to a 1:1 binding equation. Variations in maximum signal is partly due to differences in coating density between the surfaces.*

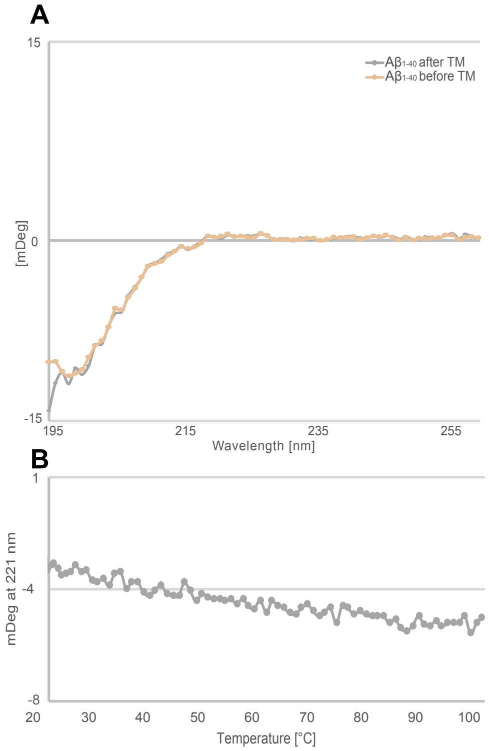

Figure S5. *Circular dichroism spectroscopy on $A\beta_{1\text{–}40}$.* ***(A)*** *Spectra between 195-260 nm for $A\beta_{1\text{–}40}$ before and after heating.* ***(B)*** *Ellipticity at 221 nm during variable temperature measurement for $A\beta_{1\text{–}40}$.*

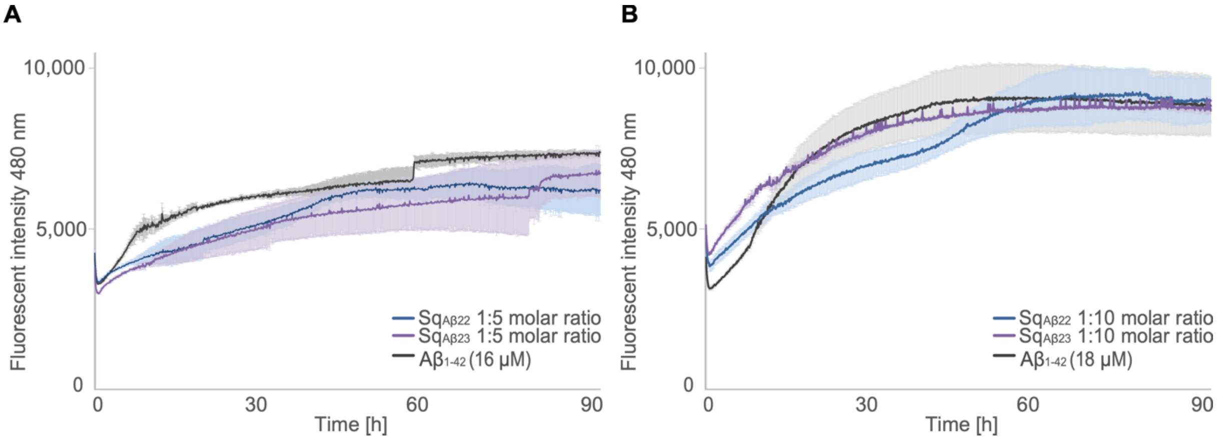

Figure S6. (A) *ThT fluorescence for 16 μM $A\beta_{1\text{–}42}$ (grey) and for 20 μM $A\beta_{1\text{–}42}$ + 4 μM $Sq_{A\beta22}$ (blue) or $Sq_{A\beta23}$ (purple) (1:5 molar ratio), respectively.* ***(B)*** *ThT fluorescence for 18 μM $A\beta_{1\text{–}42}$ (grey) and for 20 μM $A\beta_{1\text{–}42}$ + 2 μM $Sq_{A\beta22}$ (blue) or $Sq_{A\beta23}$ (purple) (1:5 molar ratio), respectively. The shaded areas represent respective standard deviation.*
